# Systematic Identification of the Serine Protease Family (StSPs) and Functional Characterization of the Secretory Protein StSP8-4 for Pathogenicity in *Setosphaeria turcica*

**DOI:** 10.3390/biology15010057

**Published:** 2025-12-28

**Authors:** Qihui Zhou, Xiaodong Gong, Jingjing Zhang, He Zhou, Mengfang Zhu, Bin Hu, Jingao Dong, Yuwei Liu, Shouqin Gu

**Affiliations:** 1 College of Life Sciences, Hebei Agricultural University, Baoding 071000, China; zhouqihui122@126.com (Q.Z.);; 2 State Key Laboratory of North China Crop Improvement and Regulation, Baoding 071000, China; 3 Hebei Bioinformatic Utilization and Technological Innovation Center for Agricultural Microbes, Baoding 071000, China; 4 Institute of Medical Science, The University of Aberdeen, Aberdeen AB24 3FX, UK; 5 College of Plant Protection, Hebei Agricultural University, Baoding 071000, China

**Keywords:** serine proteases, *Setosphaeria turcica*, phylogenetic analysis, qRT-PCR, pathogenicity

## Abstract

Serine proteases represent a significant family of proteolytic enzymes, characterized by their serine-dependent catalytic mechanism. These enzymes are integral to various biological processes, including fungal growth, development, and pathogenicity. Despite their importance, the sequence characterization and expression patterns of this protein family in *Setosphaeria turcica* are not yet fully characterized and remain underexplored. A total of 74 putative serine protease family proteins (StSPs) were identified in *S. turcica* and classified into 12 subfamilies based on phylogenetic analysis. Structural domain analysis revealed that 24 StSPs contain signal peptides, of which five were experimentally validated as secretory proteins through yeast secretion assays. Expression profiling using RNA-seq data demonstrated that *StSPs* exhibit distinct expression patterns across different developmental and infection stages, with 61 genes showing high expression during critical infection phases. The expression levels of nine genes were validated via qRT-PCR, and the results were consistent with the RNA-seq data. Among these proteins, *StSP8-4* demonstrated elevated expression during the course of fungal infection. Functional characterization of *StSP8-4* OE and RNAi strains revealed that this gene plays a crucial role in maintaining fungal pathogenicity, although silencing did not impair conidium or hyphal development. These findings provide valuable insights for further research on serine protease genes in *S. turcica*.

## Simple Summary

Northern corn leaf blight, a global foliar disease caused by *Setosphaeria turcica*, poses a serious threat to corn yield and quality. Current control strategies rely mainly on breeding resistant varieties and applying chemical agents. However, frequent variation in the pathogen often leads to rapid loss of cultivar resistance, while the emergence of pesticide resistance results in increased chemical usage, causing environmental pollution. This study investigates a key enzyme family in *S. turcica*—serine proteases—to elucidate their genetic composition and functions. We identified 74 genes in this family and classified them into 12 subfamilies, with five confirmed as secretory proteins. Most genes showed high expression during infection, particularly *StSP8-4*, which exhibited significantly elevated expression during pathogenesis. Functional experiments demonstrated that this gene is essential for fungal pathogenicity without affecting normal growth. These findings provide new insights into the pathogenic mechanisms of northern corn leaf blight and offer potential value for developing environmentally friendly disease control strategies.

## 1. Introduction

Northern corn leaf blight, caused by *Setosphaeria turcica*, is a fungal disease that significantly affects maize yield and quality [1]. The pathogen spreads by producing conidia, which germinate to form appressoria that subsequently infect maize leaves [2]. This disease induces wilting in the leaves surrounding the infection site and leads to the formation of black or brown spike-shaped lesions [3]. These lesions impair photosynthetic capacity of maize, and in severe cases, can result in plant death, thereby significantly reducing maize yields and posing a serious threat to maize production [4].

Serine proteases constitute a significant enzyme family, characterized by the nucleophilic serine present in their active site. This serine residue acts on the carbonyl group of the peptide bond in the substrate, resulting in the formation of an acyl-enzyme intermediate [5]. The activity of these enzymes is regulated by various factors, such as the binding of protease inhibitors, self-degradation, and enzymatic cascade reactions [6]. Serine proteases are widely distributed across various species, including viruses, bacteria, and eukaryotes, and can be categorized into several types, such as trypsin-like, chymotrypsin-like, elastase-like, and kininases [7]. The nucleophilicity of the catalytic serine typically depends on a catalytic triad consisting of Asp, His, and Ser residues. Enzymes utilizing this structure include trypsin, chymotrypsin, prolyl oligopeptidases, and ClpP peptidases, all of which catalyze the hydrolysis of peptide bonds. In addition, some serine proteases employ simpler dyad mechanisms, such as D-Ala-D-Ala carboxypeptidase and Lon peptidase, which rely on a Ser-Lys pair [8]. In the MEROPS database, which represents the largest curated collection of proteases, serine proteases comprise over 33% of all characterized proteolytic enzymes [9]. This superfamily is classified into 54 phylogenetically distinct families, based on their substrate recognition patterns and the structural architecture of their catalytic sites [10]. Among these families, the PA family stands as the largest serine protease superfamily, predominantly expressed in eukaryotes, while the SB and SC families are more representative of other organisms [11]. The human serine protease family is a large and diverse group of proteins comprising approximately 180 members, including nuclear pore proteins, lactoferrin, and type II transmembrane serine proteases (TTSPs). Recent studies have underscored the pivotal roles that serine proteases play in the initiation and progression of various human diseases [12].

Serine proteases play extensive physiological roles in living organisms and are involved in various key life processes [13]. In metabolism, serine proteases such as trypsin are responsible for degrading dietary proteins, breaking them down into absorbable amino acids to maintain the body’s nutritional supply [14]. In immune regulation, serine proteases participate in the amplification and modulation of immune signals by activating the complement system and cleaving cytokine precursors, thereby aiding in the recognition and clearance of pathogens [15]. Within cells, these enzymes also degrade abnormal or redundant proteins, maintain intracellular protein homeostasis, and regulate crucial life activities such as cell proliferation, differentiation, and apoptosis through specific substrate cleavage in signal transduction [16].

The role of serine proteases in the occurrence and progression of human diseases has been extensively studied [17]. Serine proteases are not only key enzymes in the basic metabolism of pathogenic fungi but also important virulence factors. For instance, TMPRSS4 facilitates influenza pathogenesis and cancer metastasis by cleaving hemagglutinin; TMPRSS2 is not only exploited by SARS-CoV-2 to enhance infectivity [18], but its overexpression and gene rearrangements also significantly promote the development of prostate cancer [19]. Additionally, serine proteases have been identified as critical pathogenic factors in *Entamoeba histolytica* [20], hepatitis C and influenza viruses [21], as well as in *Aspergillus fumigatus*, which triggers asthma [22].

In plant pathogen systems, serine protease-like effectors have also been preliminarily found to participate in the pathogenic process, though their specific functions and mechanisms of action remain to be further elucidated. For example, the Pat-1 protease in *Clavibacter michiganensis* ssp. is a key virulence factor causing bacterial canker in tomatoes [23]; Chp-7 in *C. michiganensis* is essential for eliciting host hypersensitive response [24]; and in *Phytophthora infestans*, serine protease activity is significantly positively correlated with infectivity [25]. Moreover, pathogenic fungi often utilize serine proteases to directly degrade host immune components. For instance, *Fusarium oxysporum* secretes Sep1, which works in concert with the metalloprotease Mep1 to degrade tomato chitinase [26]; *Verticillium dahliae*’s SSEP1 can hydrolyze cotton chitinase [27]. In *Valsa mali*, the serine protease VmSpm1 can be secreted into host cells and interfere with the JA signaling pathway by interacting with host proteins, thereby suppressing the plant’s defense response [28]. However, to date, no studies have characterized the serine protease family in *S. turcica.*

Previous studies in our laboratory have shown that serine proteases genes are significantly upregulated during early infection and that StSP8-4 is the sole differentially expressed secreted protein identified in proteomic screening. We hypothesized that this particular protease plays a critical role in the pathogenicity of *S. turcica*. In this study, we conducted a comprehensive analysis of the serine protease gene family in the *S. turcica* genome, elucidating its phylogenetic relationships and analyzed the protein structures and physicochemical properties of the family members. We also investigated the expression patterns of the genes via RNA-Seq data and qRT-PCR technology. The protein secretion activity was assessed using the TTC reduction assay. Moreover, to explore the role of serine proteases in the growth, development, and pathogenicity of the pathogen, we generated *StSP8-4* overexpression (OE) and RNA interference (RNAi) strains. This study provides a foundational understanding of the function and mechanism of serine proteases in *S. turcica*.

## 2. Materials and Methods

### 2.1. Strains and Culture Condition

The wild-type (WT) *S. turcica* strain 01–23 was cultured on potato dextrose agar (PDA; 20% potato extract, 2% glucose, 1.5% agar) at 25 °C for 8–10 days. The strain YTK12 was grown in yeast extract peptone dextrose medium (YPD; 1% yeast extract, 2% peptone, 2% glucose, 0.003% adenine sulfate, 2% agar) at 30 °C. *Escherichia coli* DH5α (Sangon Biotech, Shanghai, China) was used for plasmid propagation. All pathogenicity assays were performed using the maize variety B73, with plants cultivated in a greenhouse under controlled conditions (20 ± 3 °C, 16 h light/8 h dark cycle) [29].

### 2.2. Identification and Classification of StSPs

Pfam accession numbers corresponding to different StSPs families were retrieved from the MEROPS peptidase database (https://www.ebi.ac.uk/merops/, (accessed on 20 January 2025)). Subsequently, hidden Markov model (HMM) profiles for these StSPs were downloaded from the Pfam database (http://pfam.xfam.org/, (accessed on 20 January 2025)) [30]. These Hidden Markov Model (HMM) profiles were then used to screen the *S. turcica* proteome with HMMER 3.3.2 software (http://hmmer.org, (accessed on 22 January 2025)) with a threshold of E < 1.0 × 10^−5^ [31]. The protein sequences of candidate were subsequently retrieved based on their gene IDs from the JGI database (https://mycocosm.jgi.doe.gov, (accessed on 22 January 2025)).

### 2.3. Phylogenetic Analysis of StSPs

Using multiple sequence alignment results of all identified serine protease proteins, phylogenetic trees were constructed using the maximum likelihood method with 1000 bootstrap replicates. Sequence alignment was performed with ClustalW2 (http://www.clustal.org/clustal2, (accessed on 25 January 2025)) [32]. The alignment results were then imported into MEGA 6.0 software and visualized using the iTOL v6.7 online platform (https://itol.embl.de/, (accessed on 25 January 2025)) [33].

### 2.4. Analysis of Gene Structure and Protein Physicochemical Properties

Gene structure analysis of *StSPs* was performed by comparing genomic DNA sequences with corresponding coding sequences obtained from the JGI database. Gene structure visualization was conducted using the Gene Structure Display Server 2.0 (http://gsds.gao-lab.org/, (accessed on 28 January 2025)) [34].

For protein characterization, StSP amino acid sequences were analyzed with the EXPASy ProtParam tool (http://www.expasy.org/, (accessed on 30 January 2025)). The analysis provided multiple physicochemical parameters including: molecular weight, amino acid count, theoretical isoelectric point (pI), amino acid composition, and instability indices.

### 2.5. Motif and Structural Analysis of StSPs

Conserved motifs in the serine protease family were identified using Multiple Em for Motif Elicitation (MEME 5.5.9; https://meme-suite.org/meme/, (accessed on 15 February 2025)) [35]. The analysis was performed with the following parameters: zero or one motif occurrence per sequence, motif width ranging from 6 to 50 amino acids, maximum of 18 conserved segments, and an E < 1.0 × 10^−20^ for motif identification threshold [36]. Domain architectures were subsequently analyzed using SMART (https://smart.embl.de/, (accessed on 15 February 2025)).

### 2.6. RNA-Seq Experiment and Analysis

Leveraging our previously published transcriptome datasets, we performed comprehensive gene expression profiling of *S. turcica* throughout its developmental cycle and host infection process. Gene expression levels were subsequently visualized through heatmap clustering using the pheatmap package in R (v4.2.0) [4].

### 2.7. Total RNA Extraction and Real-Time Quantitative PCR (qRT-PCR)

Total RNA was extracted using the Super Plant Genomic DNA Kit (TIANGEN Biotech, Beijing, China), and the integrity of the RNA was examined by 1.0% agarose gel electrophoresis. cDNA was synthesized with the M5 Super Plus qPCR RT Kit with gDNA remover (Thermo Fisher Scientific, Waltham, MA, USA) following established protocols [37]. Specific primers were designed using Primer 3.0 (https://primer3.org/, (accessed on 27 February 2025)) (sequences provided in Table S1). The highly conserved *Tubulin* gene served as the internal reference. Quantitative reverse transcription PCR (qRT-PCR) was performed to analyze serine protease expression levels using cDNA templates from three infection time points (0 h, 24 h, and 72 h). The experiment was performed with three biological replicates and three technical replicates. Amplification was carried out using the PerfectStart™ Green qPCR SuperMix Kit (Thermo Fisher Scientific, Waltham, MA, USA) on an ABI Prism 7500 Fast Real-Time PCR system (Thermo Fisher Scientific, Waltham, MA, USA) following the manufacturer’s protocol. Relative gene expression was calculated using the 2^−ΔΔCt^ method and statistically analyzed with GraphPad Prism 6.0.1 software using Tukey’s test (*p* < 0.05) [38].

### 2.8. Yeast Secretion Activity Assay

Signal peptide prediction of serine proteases was performed using SignalP 6.0 (https://services.healthtech.dtu.dk/services/SignalP-6.0/, (accessed on 10 March 2025)). The predicted signal peptide sequences were cloned into the EcoR I and Xho I restriction sites of the yeast expression vector pSUC2t7M13ori (pSUC2) and subsequently transformed into the secretion-deficient yeast strain YTK12 for heterologous expression of pSUC2-*StSp*-SP fusion proteins. Transformants were screened on CMD-W medium (0.67% YNB, 0.075% W dropout supplement, 2% sucrose, 0.1% glucose, and 2% agar) and YPRAA medium (1% yeast extract, 2% peptone, 2% raffinose, and 2 μg mL^−1^ antimycin A), with pSUC2-*Avr1b* and pSUC2-*mg87* serving as positive and negative controls, respectively. Invertase activity was assayed with 2,3,5-triphenyltetrazolium chloride (TTC) [39].

### 2.9. Generation of StSP8-4 Overexpression and RNAi Strains

The recombinant vector pBARKS1-*StSP8-4*-*GFP* was constructed by fusing the StSP8-4 cDNA sequence to the GFP coding sequence (CDS) in the pBARKS1 vector, with removal of the stop codon to enable fusion protein expression. Specific primers were designed for both sense and antisense strands to generate the RNAi vector pSilent-*StSP8-4* targeting the *StSP8-4* CDS. Both pBARKS1-*StSP8-4*-*GFP* and pSilent-*StSP8-4* vectors were introduced into *S. turcica* via PEG-mediated transformation. Protoplast preparation and transformation procedures followed established methods [40]. Transformants were selected on PDA plates supplemented with either hygromycin B (for RNAi strains) or glufosinate (for overexpression strains). Successful generation of StSP8-4 overexpression and RNAi strains was verified by qRT-PCR.

### 2.10. Appressorium Development Observation

Hyphal and conidial suspensions were prepared from both WT and transformed strains following five days of culture growth. The suspensions were diluted and evenly spread on water agar plates overlaid with sterile cellophane. Cultures were incubated at 25 °C in darkness for 3, 12, 24, or 48 h. Microscopic examination was performed at each time point to document appressoria formation and hyphal development.

### 2.11. Growth Rate Assays

Both WT and transformed strains were cultured on potato dextrose agar (PDA) at 25 °C. Colony growth was monitored daily, with diameter measurements recorded at 24-h intervals for seven consecutive days. Growth rates were calculated based on diameter changes. The experiment was performed in triplicate.

### 2.12. Pathogenicity Assays

Pathogenicity assays were conducted when maize plants reached the 4–6 leaf stage. Mycelial suspensions were prepared by scraping 10-day-old PDA cultures of both WT and transformed strains, followed by resuspension in sterile water. Maize leaves at the 4–6 leaf stage were inoculated with 200 μL of conidial suspension per strain. Inoculated plants were initially maintained in a humid chamber at 25 °C in complete darkness for 24 h, then transferred to a growth chamber with a 12 h light/12 h dark photoperiod. Disease progression was documented photographically upon lesion appearance, followed by quantitative measurement of lesion areas [40].

## 3. Results

### 3.1. Identification, Genomic Location, and Phylogenetic Analysis of StSPs

The serine protease family is highly diverse, with 54 subfamilies currently cataloged in the MEROPS database. In this study, a total of 74 serine proteases belonging to 12 subfamilies, including S8, S9, S10, S11, S12, S15, S24, S26, S28, S41, S53, and S54, were identified and distributed across 25 scaffolds of the *S. turcica* genome. The nomenclature of the genes was determined by their subfamilies and the positions of their respective scaffolds (see Table S2).

**Figure 1:**
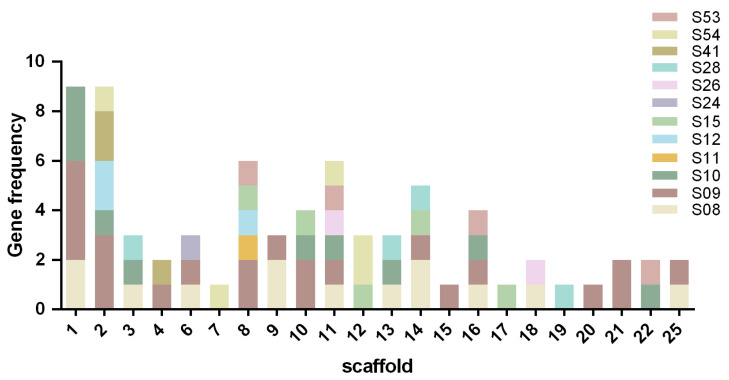
Distribution of 74 StSPs genes across *S. turcica* scaffold.

The distribution of serine protease genes across scaffolds was uneven (Figure 1). Scaffolds 1 and 2 were found to contain the highest number of genes (9 each), followed by scaffold 8 (6 genes), while only one gene was present in scaffolds 7, 15, 17, 19 and 20. The distribution of the subfamilies was as follows: the S9 subfamily, which was the most abundant, was present on all scaffolds except 7, 12, 17 and 19; the S8 subfamily, comprising 13 genes, spanned scaffolds 1, 3, 6, 9, 13, 14, 16, 18 and 25; the S26 subfamily genes were located on scaffolds 6, 11 and 18; and the S11 subfamily was exclusively found on scaffold 8.

**Figure 2:**
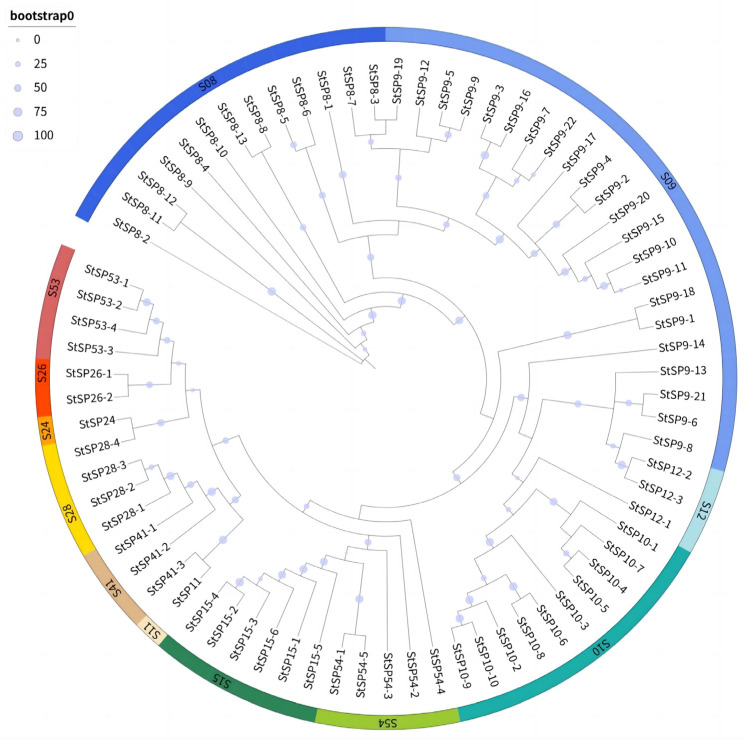
Phylogenetic relationships among StSPs subfamilies. The tree was constructed using the maximum likelihood method with 1000 bootstrap replicates, based on full-length putative amino acid sequences of StSPs aligned by ClustalW2.1. Colored arcs represent distinct StSP subfamilies.

To investigate the evolutionary relationships within the SP family of *S. turcica*, a comparison was made of 74 full-length protein sequences, and a rootless radial phylogenetic tree was constructed. The analysis revealed that all members of the serine protease family exhibited strong phylogenetic clustering, indicating structural conservation (Figure 2). The analysis revealed the presence of single-gene members within both the S11 and S24 subfamilies. Notably, StSP11 formed a monophyletic group with the *StSP41-3*, while StSP24 exhibited a close evolutionary association with the *StSP28-2*. The observed clustering patterns suggest the presence of potential common evolutionary origins between these single-gene families and their respective subfamilies.

### 3.2. Structural and Functional Characterization Analysis of StSPs

To characterize the structures of the StSP genes, the coding sequences (CDSs) of all 74 family genes were aligned with their corresponding genomic DNA. This was done in order to determine exon-intron organization. The analysis revealed that 15 genes (20%) were intronless, while the remaining exhibited variable intron numbers (Supplementary Figure S1). This structural diversity suggests an evolutionary flexibility in gene architecture among members of the StSP family. The 74 StSPs displayed considerable variation in their physicochemical properties, with protein lengths ranging from 110 to 1034 amino acids (molecular weights ranging from 11.3 to 112.65 kDa) and isoelectric points spanning 4.41 to 10.21.

The stability predictions classified 64.5% of the proteins as stable. Charge distribution analysis demonstrated that 77.6% were positively charged, 17.1% were negatively charged, and 5.3% were neutral. Hydrophobicity analysis revealed that the majority of the members were predominantly hydrophilic, although six exhibited hydrophobic properties, suggesting potential membrane association. The aliphatic indices exhibited a range from 63.34 to 114.22, as detailed in Table S2.

A comprehensive analysis of the top 10 conserved motifs was performed, revealing distinct evolutionary patterns across StSPs subfamilies (Supplementary Figure S2). The S9 subfamily was found to uniquely harbor motif 1, while the S15 subfamily possessed motifs 2, 6, and 10 in a specific manner. The S10 subfamily was characterized by the exclusive presence of motifs 3, 4, 5, and 7. Notably, motif 8 was shared between the S8 and S53 subfamilies, suggesting potential functional conservation or common evolutionary origin. Similarly, motif 9 was found in both the S9 and S15 subfamilies, indicating a close phylogenetic relationship. These motif distributions provide molecular evidence for both subfamily specialization and evolutionary connections within the StSP family.

**Figure 3:**
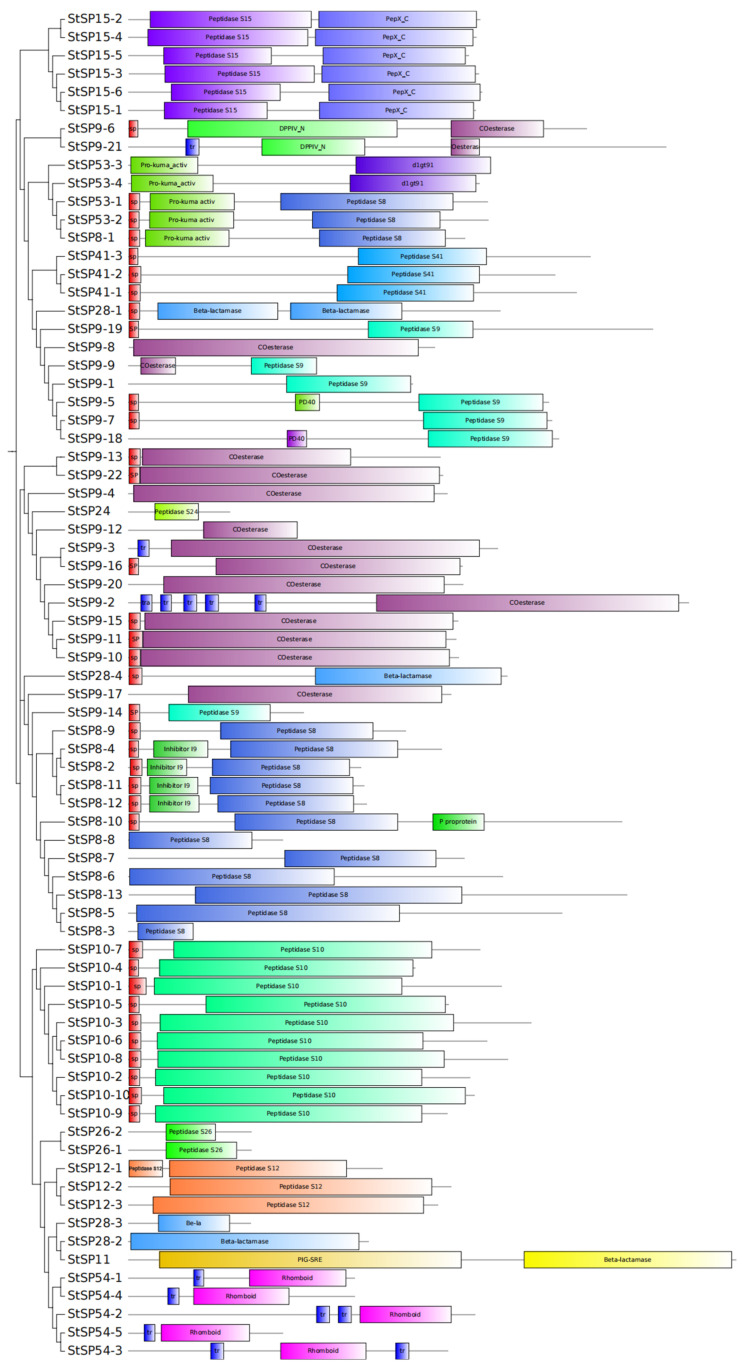
Conserved structural domains of StSPs. A total of 74 conserved StSPs domains were analyzed using SMART. Differently colored boxes represent distinct structural domains and indicate their relative positions.

Structural domain analysis revealed distinct organizational patterns among StSP subfamilies (Figure 3). The S10, S12, S24, S26, and S41 subfamilies each exhibited unique domain architectures, while other subfamilies displayed specialized combinations of domains. Specifically, the S28 contained only the β-lactamase domain; while the S54 subfamily was characterized by the rhomboid domain; The S11 subfamily included both β-lactamase and PIG-SRE domains; and the S15 subfamily combined peptidase S15 with PepX_C domains. The S53 subfamily uniformly contained the Pro-kuma activistic domain, with two members additionally possessing peptidase S8 domains and two containing d1gt91 domains. The S09 subfamily exhibited alternative domain configurations, including either an esterase or peptidase S9 domain. The S8 subfamily consistently maintained the peptidase S8 domain coupled with either I9 inhibitor or fn3-5 domains. Notably, the presence of signal peptide domains in certain members suggests extracellular secretion, while the presence of transmembrane domains suggest membrane localization potential. This collective evidence demonstrates the manner in which domain architecture underpins functional specialization across StSP subfamilies.

### 3.3. Expression Analysis of StSPs at Different Stages of Development and Infection

**Figure 4:**
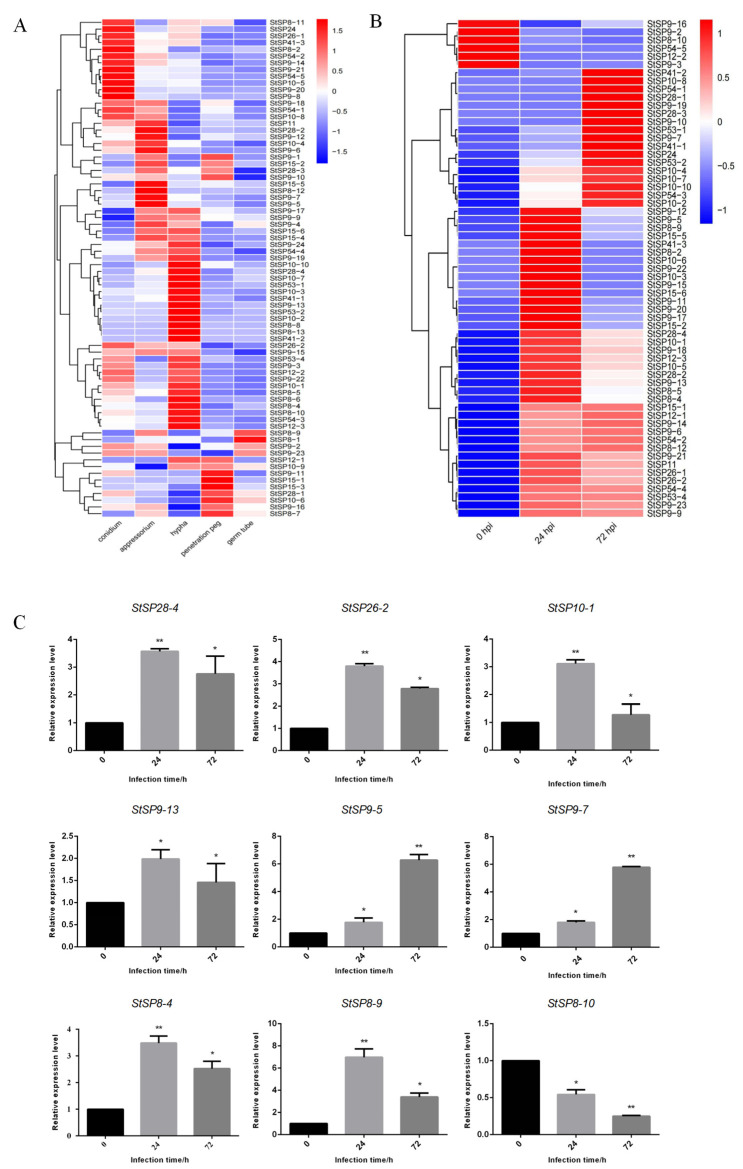
Expression patterns of *StSPs* family genes in response to infection and developmental processes. (**A**) Heatmap showing expression profiles of *StSPs* genes during fungal development stages: conidium, appressorium, germ tube, penetration peg, and hypha. (**B**) Heatmap of *StSPs* gene expression in maize infected with *S. turcica* at 0, 24, and 72 h post-inoculation (hpi). (**C**) qRT-PCR analysis of selected *StSPs* genes with *Tubulin* as an internal control. Data represent mean ± SD (*n* = 3). Statistical significance was analyzed using two-tailed Student’s *t*-test (* *p* < 0.05; ** *p* < 0.01).

To investigate the function of StSPs genes, we analyzed their expression profiles at various growth stages and infection periods using RNA-seq data. This identified 74 differentially expressed during the growth and development of *S. turcica* (Figure 4A). Among these, 12 genes were highly expressed during the conidium stage, belonging to subfamilies S8, S9, S10, S24, S26, and S54. In the hyphal stage, 17 genes from subfamilies S8, S9, S10, S12, S28, S41, S53, and S54 showed increased expression. Seven genes were upregulated during the penetration peg stage, and six during the appressorium stage, with *StSP8-1* uniquely expressed in the germ tube stage. Several genes had elevated expression across multiple periods; notably, six genes from subfamilies S09, S10, S11, and S54 were highly expressed in both conidium and appressorium stages, while four genes from S9, S15, and S28 peaks during conidium and penetration peg stages. Differential expression of *StSP8-9* was noted during the appressorium and germ tube stages, whereas *StSP12-1* and *StSP10-9* increased during the penetration peg and germ tube stages. Additionally, *StSP26-2* and *StSP9-15* showed significant variation across the conidium, appressorium, and hyphal stages, and two S09 genes (*StSP9-2* and *StSP9-23*) had elevated expression in both hyphal and germ tube stages. During maize infection, 61 genes showed significant expression fluctuations (Figure 4B). 6 genes peaked at 0 hpi, followed by a decrease, while 17 genes peaked at 72 hpi, and 24 genes peaked at 24 hpi before declining. Fourteen genes continued to increase after 24 hpi.

To validate the RNA-seq data, we conducted qRT-PCR on nine selected serine protease genes (*StSP8-4*, *StSP10-1*, *StSP26-2*, *StSP9-5*, *StSP9-13*, *StSP8-9*, *StSP9-7*, *StSP8-10*, and *StSP28-4*) at different infection periods, using *Tubulin* as an internal reference (Figure 4C). The qRT-PCR results confirmed that *StSP9-5* expression increased over time, while *StSP8-10* decreased. Other genes initially declined but remained higher than at 0 hpi, aligning with RNA-seq data and validating its accuracy and reliability.

### 3.4. Prediction and Validation of StSPs Secretion Activity

Structural domain analysis revealed that 24 StSPs possess signal peptide structures, representing 31% of the total StSPs population. This finding indicates that these proteins have the potential to be secreted, thereby functioning outside the cell. Furthermore, analysis of the signal peptide sequences revealed that the length of the signal peptides ranged from 15 to 30 amino acids (AAs), with the majority comprising 20 AAs. The signal peptide sequences of StSPs contained all 20 standard amino acids, with prediction probabilities ranging from 0.9731 (StSP9-6) to 0.1973 (StSP10-5) (Table 1).

**Figure 5:**
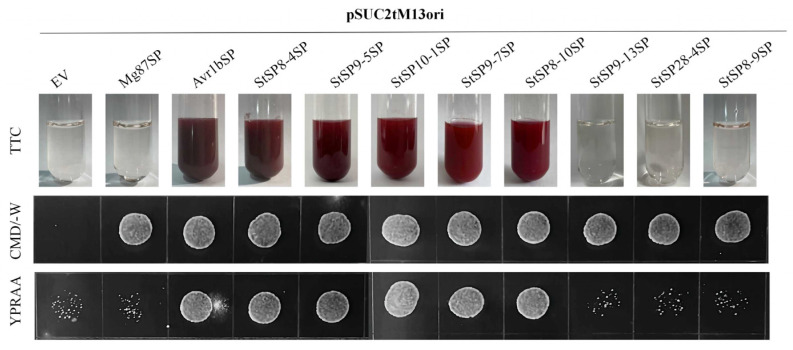
Functional evaluation of signal peptides in 8 StSPs. The secretion function of the signal peptides of candidate effectors was confirmed using a yeast secretion system. The function of signal peptides from 8 candidate effectors was identified. Yeast transformants were cultured on CMD-W medium, sucrose plates, and YPRAA medium. The invertase activity was detected by reduction of TTC to the insoluble, red-colored 1,3,5-triphenylformazan (TPF). Transformed cells were collected in tubes to detect invertase activity. The negative control was the YTK12 strain carrying the signal peptide of *Mg87*, and the positive control was the YTK12 strain carrying the signal peptide of *Avr1b*.

To validate the predicted signal peptide functions, eight serine protease genes that were highly expressed during infection were selected: StSP8-4, StSP8-9, StSP8-10, StSP9-5, StSP9-7, StSP9-13, StSP10-1 and StSP28-4. Samples were cultivated on YPRAA medium and subjected to TTC staining. The results revealed that five proteases, namely StSP8-4, StSP9-5, StSP10-1, StSP9-7, and StSP8-10 exhibited both normal proliferation capacity and the ability to catalyze the conversion of TTC into red reaction products, thus confirming their secretory activity (Figure 5).

### 3.5. StSP8-4 Exerts No Influence on Development but Is Involved in the Pathogenicity of S. turcica

**Figure 6:**
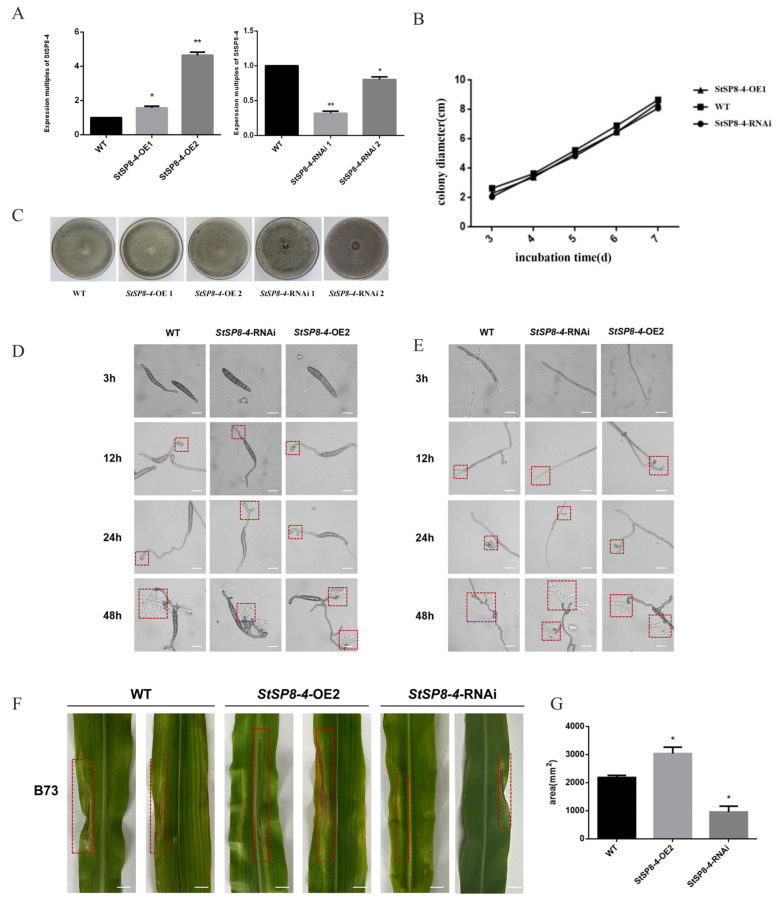
The *StSP8-4* gene has no significant effect on growth and development but affects the pathogenicity of *S. turcica*. (**A**) The expression levels of *StSP8-4* in various strains were quantified by qRT-PCR. Data represent mean ± SD (*n* = 3) were analyzed using a two-tailed Student’s *t*-test (* *p* < 0.05; ** *p* < 0.01). (**B**) *StSP8-4*-OE and *StSP8-4*-RNAi strains were created and inoculated together on PDA medium. Phenotypes were observed after 14 days. (**C**) Growth rates of WT, *StSP8-4*-OE2, and *StSP8-4*-RNAi strains were calculated using the crossover method and presented as histograms. (**D**) Hyphal development was observed by microscopy at 3 h, 12 h, 24 h, and 48 h after incubating WT, *StSP8-4*-OE2, and *StSP8-4*-RNAi strains at 25 °C in the dark for approximately 5 days, The area within the red dashed box represents the appressorium (scale bar = 10 μm). (**E**) Conidial development was recorded microscopically at the same time points under identical culture conditions, The area within the red dashed box represents the appressorium (scale bar = 10 μm). (**F**) Mycelia of WT, *StSP8-4*-OE2, and *StSP8-4*-RNAi strains cultured on PDA for over 10 days were inoculated onto B73 corn plants at the 4–6 leaf stage to assess virulence. The area within the red dashed box indicates the lesion. Scale bar = 1 cm. (**G**) Lesion areas were quantified using ImageJ software 1.8.0.345. Data represent mean ± SD (*n* = 3) were analyzed using a two-tailed Student’s *t*-test (* *p* < 0.05).

To investigate the function of the *StSP8-4* gene, both overexpressing and RNAi strains were generated. qRT-PCR analysis revealed that *StSP8-4* expression levels were elevated 1.6-fold in *StSP8-4*-OE 1 and 4-fold in *StSP8-4*-OE 2 compared to WT, and *StSP8-4*-RNAi 1 strains exhibited only 30%, Expression levels in the StSP8-4-RNAi strains were reduced to 30% (RNAi 1) and 80% (RNAi 2) of the WT level (Figure 6A). We selected the *StSP8-4*-OE 2 and *StSP8-4*-RNAi 1 strains for further study based on these results.

No significant differences were observed in growth rates or colony morphology between the *StSP8-4*-OE 2, *StSP8-4*-RNAi 1, and WT strains after 14 days of culture on PDA media (Figure 6B,C). Microscopic examination revealed comparable growth rates and morphology in mycelia and conidia among WT, *StSP8-4*-OE 2, and *StSP8-4*-RNAi 1 strains (Figure 6D,E). These findings suggest that *StSP8-4* does not play a regulatory role in fungal growth and development.

Pathogenicity assays showed distinct lesion formation on maize leaves. Both the WT and *StSP8-4*-OE 2 strains produced well-defined lesions, whereas *StSP8-4*-RNAi 1 strains formed significantly smaller lesions (Figure 6F). Subsequent analysis of the lesions areas revealed that, compared to the WT strains, *StSP8-4*-OE 2 developed 50% larger lesions, while *StSP8-4*-RNAi 1 showed 60% smaller lesions. These findings indicates that *StSP8-4* positively regulates fungal pathogenicity, underscoring its role in the pathogenicity of *S. turcica* (Figure 6G).

## 4. Discussion

Serine proteases are a class of widely distributed and functionally diverse class of proteases found in living organisms [41]. They are classified into several subfamilies based on their amino acid sequences and structural characteristics, with a common characteristic being their ability to hydrolyze peptide bonds at serine residues within proteins [42]. Currently, the MEROPS database lists 54 serine protease subfamilies, which exhibit significant variations in distribution across different species [9]. For instance, serine proteases in prokaryotes, are predominantly found in the subfamilies S1, S8, S12, S14, and S16 [20]. It was initially thought that serine proteases were less abundant in plants [43]. However, genome analyses of *Arabidopsis thaliana* and wheat have revealed the presence of 13 serine protease subfamilies [17]. In fungi, 21 serine protease subfamilies have been identified, with 14 of these subfamilies shared between plants and animals [44]. In this study, we systematically identified 12 serine protease subfamilies in the genome of *S. turcica*, comprising 74 StSPs proteins. The majority of these proteins belong to the S8, S9, or S10 subfamilies. These findings suggest that the diversity of serine proteases is the result of long-term evolutionary selection and functional differentiation, providing a molecular foundation for species adaptation to diverse ecological and biological environments.

Serine proteases play a critical role in a variety of physiological processes, including metabolic regulation, cell signalling, defense mechanisms, and growth and development. The different subfamilies of serine proteases have distinct structural domains and substrate specificities, which enable them to perform a variety of functions in different biological contexts [45]. The S9 subfamily, also known as prolyl oligopeptidases (POPs), is a widespread and abundant group of serine proteases found in both plant and fungal genomes. POPs regulate a variety of biological functions by processing key peptide hormones [46]. In pathogenic fungi, POPs have been implicated in enhancing pathogenicity by hydrolyzing small peptides associated with host immune responses, thus facilitating immune evasion [47]. In our study, 12 proteases of the S09 family were found to be highly expressed during the appressorium stage of *S. turcica*, suggesting a potential role for these enzymes in fungal growth and development. The S8 subfamily, the second-largest group of serine proteases, includes subtilisin-like serine endopeptidases. In *A. thaliana*, members of the S8 family, such as At-SLP2 and At-SLP3 play key roles in regulating stomatal distribution, leaf development, and disease resistance [48]. Similarly, in pathogenic fungi, S8 proteases are critical virulence factors. For instance, in Magnaporthe oryzae, deletion of the subtilisin-like serine protease SPM1 significantly reduces conidial formation and autophagosome accumulation, thereby impairing pathogenicity [49]. Another S8 member, prb1, encodes a protease in Cryphonectria parasitica, and prb1 strains exhibit reduced aerial hyphal formation and decreased virulence [40]. In the present study, five genes from the S8 subfamily in *S. turcica* were found to be highly expressed during infection, suggesting their potential involvement in the pathogenic processes. The S53 subfamily, also known as sedolisins, shares structural motifs and domains with the S08 family, indicating functional similarities. Sedolisins secreted by Botrytis cinerea during infection may contribute to cell wall softening and the suppression of plant defenses [50]. The S54 family, known as rhomboid proteases, consists of intramembrane serine proteases that cleave transmembrane proteins. These proteins feature six transmembrane domains and a serine-histidine catalytic dyad [51]. In mammals, the rhomboid protein RHBDL4 facilitates the transport of TGFα from the endoplasmic reticulum (ER) to the Golgi apparatus, thereby triggering extracellular vesicle secretion and regulating ER protein homeostasis [52]. In *S. turcica*, four rhomboid proteins were identified, and their expression levels undergoing significant changes during both the infection and developmental stages. These four genes may play critical roles in growth and development, as well as in helping the fungus evade host immune responses.

Effectors are secreted proteins produced by pathogenic microorganisms during host infection. They proteins delivered into host cells through specific mechanisms, where they modulate host physiological processes, suppress immune responses, or alter cellular functions, thereby facilitating pathogen invasion and proliferation [18]. Some proteases function as effectors, directly or indirectly inhibiting plant immune responses. For instance, glycoside hydrolases (GHs) degrade polysaccharide chains in plant cell walls, disrupting structural barriers and promoting pathogen invasion [53]. These proteases not only break down plant cell walls but also release nutrients that support pathogen growth and multiplication [54]. AvrRpt2, an effector with cysteine protease activity in *Pseudomonas syringae* DC3000, is initially identified due to its ability to trigger disease resistance in *A. thaliana* plants carrying the resistance gene RPS2. AvrRpt2 can enhance the virulence of the pathogen in host plants lacking a functional RPS2 gene [55]. Proteases not only degrade target proteins during infection but also modulate intracellular signaling, cell cycle regulation, and immune responses through protein–protein interactions [38]. Furthermore, we found that StSP8-4 exhibits significant structural homology with VmSpm1 from *V. mali*, and both belong to the S8 family of secreted serine proteases. It is known that VmSpm1 suppresses plant immunity by targeting key components of the host jasmonic acid (JA) signaling pathway [28]. Given the high similarity in structure and secretion patterns, we infer that StSP8-4 is also likely to target the JA signaling pathway and play a similar immunosuppressive role in *S. turcica*. In this study, we identified StSP8-4 as a novel secreted serine protease whose expression was significantly upregulated during *S. turcica* infection. Research has shown that known virulence factors such as the septin protein StSep4 are crucial for regulating cell morphology, integrity, and basic growth in *S. turcica* [56]. In contrast, StSP8-4 did not affect normal fungal growth or development and was confirmed to be secretory. This indicates that StSP8-4 likely functions as a secreted protein exported to the extracellular environment, where it acts during host–pathogen interactions. Thus, its mode of action distinctly differs from that of conventional intracellular virulence factors. The *StSP8-4* as a potential effector protein provides new insights into the complex interactions between pathogen virulence factors and plant immunity. However, the mechanism of action of StSP8-4, particularly its specific function as a protease, remains unclear and constitutes the central focus of our subsequent research. In the next steps, we will conduct experiments focusing on two main directions: validating its enzymatic activity and analyzing the function of its potential effectors, to systematically elucidate the mode of action of StSP8-4.

## 5. Conclusions

In this study, we conducted a comprehensive analysis of the phylogenetic relationships, gene structures, conserved motifs, and expression patterns of StSPs in *S. turcica*, providing valuable theoretical insights into the functional roles of this protease family in *S. turcica*. A total of 74 putative serine protease genes were identified, and the secondary structures and physicochemical properties of each member were extensively characterized, thereby establishing a robust foundation for subsequent functional studies. In addition, the expression patterns of nine serine protease genes were determined, the secretory activity of eight serine protease signal peptides was validated, and *StSP8-4* overexpression and RNAi strains were generated. These studies provided preliminary insights into the biological functions of serine proteases in the growth, development, and infection processes of *S. turcica*.

## Figures and Tables

**Table 1:** Prediction of serine protease signal peptide.

Protein Name	ProteinID	Length	Cleavage Site	Sequence	Probability
StSP9-24	161799	15	AAA-SP	MRYSLIAALPALAAA	0.4592
StSP8-10	168208	19	AAA-SH	MRIHAWGLLSALTALAAAA	0.4057
StSP53-1	99451	19	ALA-SP	MHMLASVLLTALAAREALA	0.8439
StSP28-1	32685	19	AAA-AC	MTILTTFVAAAAAGLVAVSKA	0.3201
StSP9-7	162968	18	ACA-LT	MARYLSVAAALAATGACA	0.4781
StSP9-10	173504	20	SDA-AA	MLRKAALATALFIGLGVSDA	0.7671
StSP10-2	162332	19	ALG-QF	MRSSTKLVVAPLLAAGALG	0.7953
StSP10-4	162959	17	VLS-LS	MKLSIVNLLAATTPVLS	0.4439
StSP10-7	158789	24	VSA-RS	MDYCLPNKLLSIIAVCGLTASVSA	0.4004
StSP53-2	175288	18	GSA-AR	MKYNLLLAGLLAVGSGSA	0.4712
StSP10-10	184812	20	SHA-AA	MLTKSIATPLLALSSLVSHA	0.4375
StSP10-6	158630	21	TLA-QF	MFPFARCAALTLLFSAVPTLA	0.8758
StSP8-2	113614	15	VLA-LP	MQLSLLLALLPAVLA	0.4714
StSP9-15	37031	15	VCA-VP	MKFLSALACVPLVCA	0.4578
StSP9-5	87464	17	AQA-IT	MKTALVAAGLLLQSAQA	0.8806
StSP8-9	168109	20	VFA-TP	MKSFFAIAAGLLAACSPVFA	0.9557
StSP9-6	88934	16	TQA-IE	MRSLLLLAAALPLTQA	0.9731
StSP8-12	39300	21	IQA-AP	MQLFTRIAALAAAAAPFLIQA	0.5497
StSP9-13	166367	20	AVT-AP	MHHLLLTAAAVASLVGSAVT	0.3211
StSP12-3	1414903	18	AVA-SP	MKLVASLVCGVAALGAVA	0.5857
StSP10-5	164048	15	AAA-VA	MKVATSALLVGAAAA	0.1973
StSP10-1	134338	30	IMA-AE	MASSHPPSRWRTALLGGLVATVAWLPSIMA	0.6277
StSP28-4	109485	23	VAA-VP	MYTSRSLFSTLANCLSLATLVAA	0.5632
StSP8-4	163614	15	AAA-SP	MRYSLIAALPALAAA	0.9357

## Data Availability

The RNA-Seq data are available under accession number PRJNA1185298 on the NCBI server (http://www.ncbi.nlm.nih.gov/sra, (accessed on 20 February 2025)).

## Supplementary Materials

The following supporting information can be downloaded at: https://www.mdpi.com/article/10.3390/biology15010057/s1, Figure S1: Exon-intron structure of StSPs gene of *S. turcica*; Figure S2: Motif analysis of the StSPs gene family of *S. turcica*; Table S1: Primers used in this study; Table S2: Physicochemical properties of the serine protease family.
