# Supplementary material for: Systematic Identification of the Serine Protease Family (StSPs) and Functional Characterization of the Secretory Protein StSP8-4 for Pathogenicity in Setosphaeria turcica
Source: Biology (Basel). 2025 Dec 28;15(1):57. doi: 10.3390/biology15010057 (PMC12784987; doi:10.3390/biology15010057)
Supplement: Supplementary file 1 [file biology-15-00057-s001.zip › Supplementary Table S1.pdf]

Table S1 Primers used in this study.

Primer names	primer sequence
SP-*StSP9-5*-F	GAATTCATGAAGCTGCATTGGTG
SP-*StSP9-5*-R	CTCGAGGTCCGTGTCGAGGAAAT
SP-*StSP10-1*-F	GAATTCATGGCTTCCTCACACCC
SP-*StSP10-1*-R	CTCGAGTGCCAGAAGAAGAGGTT
SP-*StSP8-4*-F	GAATTCATGAGGTACAGCCTGAT
SP-*StSP8-4*-R	CTCGAGTGTAGCTGTTGGGCACC
SP-*StSP9-13*-F	GAATTCATGCACCACCTCCTGCT
SP-*StSP9-13*-F	GAATTCATGCACCACCTCCTGCT
SP-*StSP9-13*-R	CTCGAGGCTGGGCGGCGCAAATC
SP-*StSP28-4*-F	GAATTCATGTATACCTCACGCTC
SP-*StSP28-4*-R	CTCGAGCGTGTACATTTAGGGAA
SP-*StSP26-2*-F	GAATTCATGGCTTCCTCACACCC
SP-*StSP26-2*-R	CTCGAGTGCCAGAAGAAGAGGTT
SP-*StSP8-9*-F	GAATTCATGAAGTCCTTCTTCGC
SP-*StSP8-9*-R	CTCGAGCGCCGTCGTTGTTGAGC
SP-*StSP9-7*-F	GAATTCATGGCTCGTTACCTCAG
SP-*StSP9-7*-R	CTCGAGCCGGTGGTGAGGTCAAT
SP-*StSP8-10*-F	GAATTCATGCGCATCCACGCCTG
SP-*StSP8-10*-R	CTCGAGTGAAGACATGGTGGTCG
OE-*StSP8-4*-F	CATATGATGAGGTACAGCCTGATCGC
OE-*StSP8-4*-R	GAATTCGTGGCTAGCGAGGCGCTCCT
GFP-F	GAATTCATGGTGAGCAAGGGCGAGG
GFP-R	ATCGATGTACAGCTCGTCCATGCCGA
RNAi-*StSP8-4*-Z-F	TACGTATGGTCGTGTTCAAGAAGCACGTCAA
RNAi-*StSP8-4*-Z-R	AAGCTTCGGATCGCCTCGAGGGTCTCGTCGT
RNAi-*StSP8-4*-F-F	CCCGGGTGGTCGTGTTCAAGAAGCACGTCAA
RNAi-*StSP8-4*-F-R	GCATGCTCGATGTAGTCAACATCAGGGTGGT
Tubulin-F	GGGAACTCCTCACGGATGTTG
Tubulin-R	TAACAACTGGGCAAAGGGTCA
