# Supplementary material for: Systematic Identification of the Serine Protease Family (StSPs) and Functional Characterization of the Secretory Protein StSP8-4 for Pathogenicity in Setosphaeria turcica
Source: Biology (Basel). 2025 Dec 28;15(1):57. doi: 10.3390/biology15010057 (PMC12784987; doi:10.3390/biology15010057)
Supplement: Supplementary file 1 [file biology-15-00057-s001.zip › Supplementary Table S2.pdf]

Table S2 Physicochemical properties of the serine protease family

no.	Protein name	Protein ID	Chr	Family	Protein length	Mol. Wt (Da)	pI	Negatively charged residues	Positively charged Residues	Instability index	Stability	Aliphatic Index	GRAVY
1	StSP8-1	18922	1	S08	573	62786.57	5.47	54	41	31.19	stable	80	-0.231
2	StSP8-2	113614	1	S08	396	40561.5	8.29	28	30	31.25	stable	86.04	0.003
3	StSP8-3	63240	9	S08	110	11304.79	6.03	11	9	-10.94	stable	74.45	0.037
4	StSP8-4	163614	3	S08	533	57179.8	5.94	70	58	35.36	stable	71.28	-0.439
5	StSP8-5	165339	6	S08	738	78953.67	4.9	79	50	27.26	stable	79.27	-0.139
6	StSP8-6	99952	9	S08	637	68036.61	5.38	51	42	30.04	stable	84.91	-0.039
7	StSP8-7	25785	11	S08	572	64050.09	6.56	66	62	44.47	unstable	87.53	-0.306
8	StSP8-8	1316230	13	S08	263	28934.93	8.22	30	32	41.26	unstable	86.43	-0.272
9	StSP8-9	168109	14	S08	472	49167	5.93	43	37	33.34	stable	77.73	-0.137
10	StSP8-10	168208	14	S08	840	92438.68	5.15	125	86	38.63	stable	70.52	-0.579
11	StSP8-11	149807	16	S08	401	42167.14	5.65	35	29	27.35	stable	90.07	-0.004
12	StSP8-12	39300	18	S08	406	41624.19	5.98	34	29	27.01	stable	80.32	-0.071
13	StSP8-13	161779	25	S08	848	89230.81	5.79	65	55	29.44	stable	87.56	0.03
14	StSP9-1	87019	1	S09	484	53824.38	6.13	45	37	40.99	unstable	76.34	-0.13
15	StSP9-2	113413	1	S09	954	106302.68	7.95	90	93	36.99	stable	85.37	-0.153
16	StSP9-3	113628	1	S09	629	70500.88	7.11	78	78	36.09	stable	71.64	-0.511
17	StSP9-4	153080	1	S09	543	60399.67	5.95	63	54	52.43	unstable	77.27	-0.263
18	StSP9-5	87464	2	S09	716	79117.88	4.95	79	52	29.34	stable	74.11	-0.318
19	StSP9-6	88934	2	S09	780	87809.05	5.06	99	71	31.58	stable	75.73	-0.423
20	StSP9-7	162968	2	S09	721	79441.21	4.99	68	42	31.77	stable	74.62	-0.306
21	StSP9-8	172761	4	S09	320	35513.59	4.98	37	26	53.24	unstable	87.69	-0.139
22	StSP9-9	173504	6	S09	562	61626.24	4.95	57	42	42.1	unstable	76.41	-0.208

no.	Protein name	Protein ID	Chr	Family	Protein length	Mol. Wt (Da)	pI	Negatively charged residues	Positively charged Residues	Instability index	Stability	Aliphatic Index	GRAVY
23	StSP9-10	99416	8	S09	558	61358.44	5.1	57	41	30.29	stable	76.47	-0.294
24	StSP9-11	157444	8	S09	287	31646.69	6.14	31	27	24.81	stable	63.34	-0.428
25	StSP9-12	166367	9	S09	531	57025.05	6.63	40	38	31.21	stable	78.15	-0.112
26	StSP9-13	45067	10	S09	263	28934.93	8.22	30	32	41.26	unstable	86.43	-0.272
27	StSP9-14	37031	11	S09	561	60462.07	5.1	47	41	35.82	stable	94.03	0.018
28	StSP9-15	166658	10	S09	569	62096.18	6.36	47	43	32.38	stable.	79.96	-0.11
29	StSP9-16	168207	14	S09	733	81412.87	5.69	91	71	37.95	stable	71.19	-0.533
30	StSP9-17	149188	15	S09	892	98103.54	6.05	88	72	36.09	stable	87.77	-0.184
31	StSP9-18	149779	16	S09	570	61440.48	7.13	46	46	40.51	unstable	78.11	-0.113
32	StSP9-19	151100	20	S09	915	103753.1	5.5	132	110	39.33	stable.	74.03	-0.603
33	StSP9-20	161464	21	S09	521	56617.56	5.16	44	37	37.73	stable	76.64	-0.149
34	StSP9-21	1116034	21	S09	549	60832.62	9.02	49	58	49.04	unstable	72.17	-0.401
35	StSP9-22	161799	25	S09	535	59446.87	6.28	52	48	35.17	stable	77.68	-0.362
36	StSP10-1	134338	1	S10	635	71091.67	5.3	91	70	51.87	unstable	71.45	-0.589
37	StSP10-2	162332	1	S10	582	63904.43	5.52	51	44	31.7	stable	63.63	-0.375
38	StSP10-3	162513	1	S10	685	73935.62	5.03	66	44	37.91	stable	78.8	-0.223
39	StSP10-4	162959	2	S10	488	54409.74	5.5	52	40	41.11	unstable	75.51	-0.247
40	StSP10-5	164048	3	S10	545	61122.78	5.19	71	52	41.65	unstable	73.93	-0.422
41	StSP10-6	158630	10	S10	610	67232.72	4.55	69	40	31.61	stable	70.13	-0.331
42	StSP10-7	158789	11	S10	559	61886.38	5.49	53	44	42	unstable	80.29	-0.168
43	StSP10-8	159655	13	S10	646	71213.42	5.08	60	60	34.35	stable	73.39	-0.347
44	StSP10-9	27640	16	S10	542	60421.08	4.62	68	39	37.02	stable	70.57	-0.355

no.	Protein name	Protein ID	Chr	Fami ly	Protein length	Mol. Wt (Da)	pI	Negatively charged residues	Positively charged Residues	Instability index	Stability	Aliphatic Index	GRAVY
45	StSP10-10	184812	22	S10	589	65674.03	4.7	60	33	28.65	stable	71.41	-0.294
46	StSP11	122912	8	S11	1034	112655.17	5.62	106	81	44.08	unstable	88.38	-0.147
47	StSP12-1	153523	2	S12	432	47699.83	5.92	44	41	30.11	stable	72.04	-0.329
48	StSP12-2	162786	2	S12	549	62094.45	4.91	66	44	40.08	unstable	79.76	-0.345
49	StSP12-3	1414903	8	S12	527	58141.01	6	42	34	34.39	stable	70.97	-0.288
50	StSP15-1	91685	3	S15	591	66270.08	5.61	78	64	32.9	stable	72.77	-0.413
51	StSP15-2	23952	8	S15	599	68253.67	6.02	84	73	31.86	stable	72.55	-0.59
52	StSP15-3	101417	10	S15	596	67344.18	5.53	83	63	47.11	unstable	75.74	-0.414
53	StSP15-4	103755	12	S15	592	67125.14	5.76	77	62	38.17	stable	71.17	-0.558
54	StSP15-5	167975	14	S15	579	64306.85	5.55	75	59	42.46	unstable	82.9	-0.316
55	StSP15-6	108687	17	S15	602	67389.46	5.74	79	61	43.16	unstable	75.98	-0.41
56	StSP24	1305443	6	S24	173	19086.47	8.01	15	16	26.54	stable	114.22	0.327
57	StSP26-1	126238	11	S26	210	23180.57	8.64	19	21	34.5	stable	84.24	-0.042
58	StSP26-2	168876	18	S26	208	23878.88	5.89	33	29	46.89	unstable	69.76	-0.629
59	StSP28-1	32685	3	S28	633	68343.33	5.52	59	44	37.7	stable	88.99	-0.043
60	StSP28-2	148034	13	S28	409	45414.28	5.34	58	49	36.04	stable	79.49	-0.323
61	StSP28-3	38205	14	S28	445	48291.48	5.41	52	38	43.45	unstable	87.93	-0.243
62	StSP28-4	109485	19	S28	645	71283.02	6.12	62	55	24.65	stable	78.05	-0.264
63	StSP41-1	20011	2	S41	763	83282.45	5.67	72	62	31.85	stable	76.87	-0.237
64	StSP41-2	31448	2	S41	726	81013.76	7.21	65	65	32.77	stable	76.21	-0.24
65	StSP41-3	180239	4	S41	786	85433.23	4.45	100	57	37.14	stable	78.46	-0.264

66	StSP53-1	99451	8	S53	611	67263.33	6.42	57	52	27.86	stable	73.45	-0.35

no.	Protein name	Protein ID	Chr	Family	Protein length	Mol. Wt (Da)	pI	Negatively charged residues	Positively charged Residues	Instability index	Stability	Aliphatic Index	GRAVY
67	StSP53-2	175288	11	S53	613	66925.22	5.9	70	64	40.31	unstable	77.29	-0.357
68	StSP53-3	27753	16	S53	616	67454.62	4.98	68	44	30.61	stable	65.42	-0.527
69	StSP53-4	177490	22	S53	598	65122.88	5.25	64	47	35.37	stable	78.13	-0.31
70	StSP54-1	135683	2	S54	385	43475.19	10.11	22	38	44.04	unstable	85.12	-0.077
71	StSP54-2	142012	7	S54	590	66478.53	8.94	67	76	45.7	unstable	85.36	-0.288
72	StSP54-3	102778	11	S54	544	60075.08	9.12	36	47	51.75	unstable	82.63	-0.146
73	StSP54-4	127468	12	S54	273	29916.73	10.21	6	23	44.3	unstable	87.55	0.152
74	StSP54-5	167629	12	S54	267	28285.31	6.52	30	29	25.93	stable	81.84	-0.015

pI, isoelectric point; MW, molecular weight.
