# Supplementary figures and images for: Systematic Identification of the Serine Protease Family (StSPs) and Functional Characterization of the Secretory Protein StSP8-4 for Pathogenicity in Setosphaeria turcica
Source: Biology (Basel). 2025 Dec 28;15(1):57. doi: 10.3390/biology15010057 (PMC12784987; doi:10.3390/biology15010057)

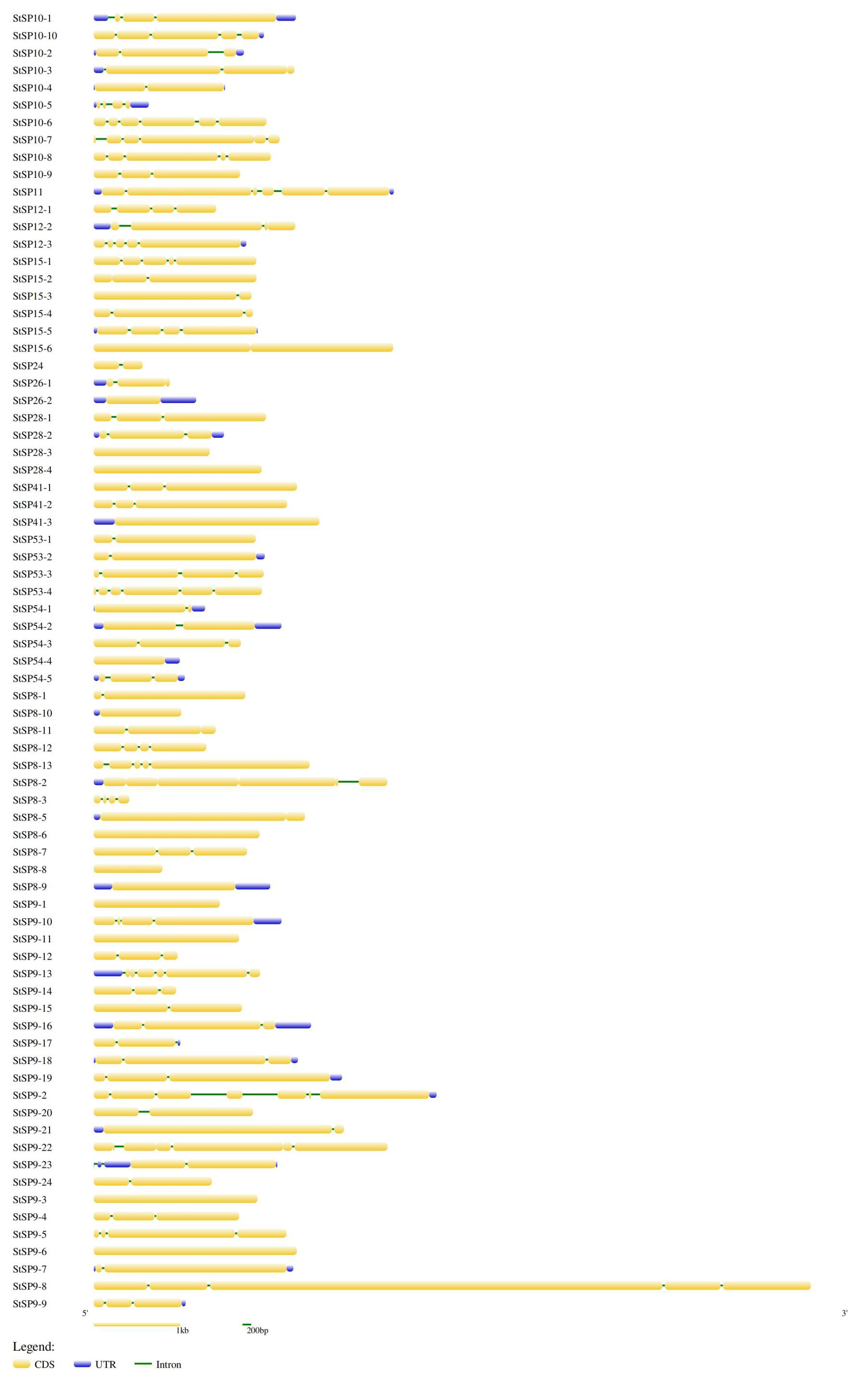

Supplement: Supplementary file 1 [file biology-15-00057-s001.zip › Supplementary Figure S1.tif]

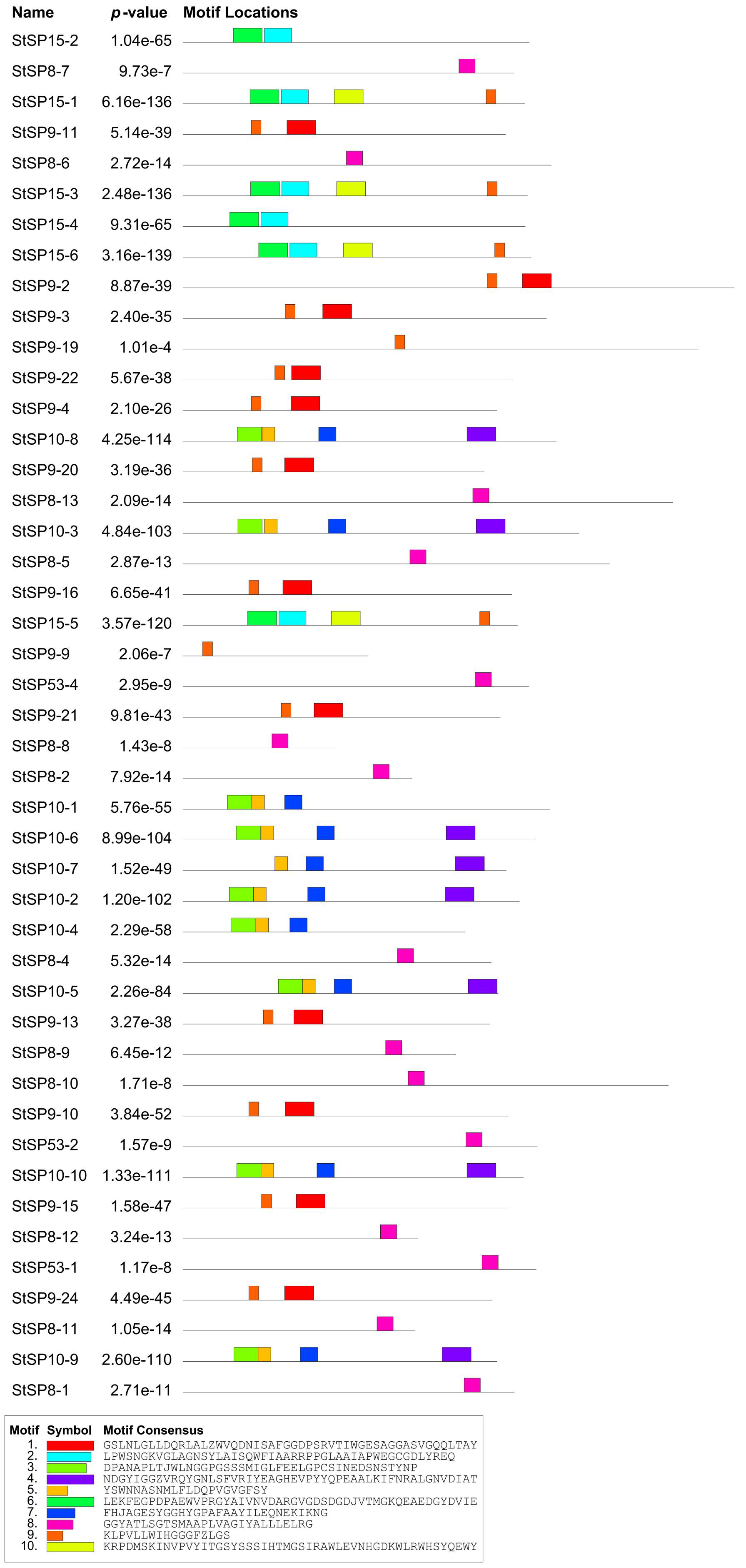

Supplement: Supplementary file 1 [file biology-15-00057-s001.zip › Supplementary Figure S2.tif]
